# Impact of the Suspension of Dental Service on Oral Health-related Quality of Life in Orthodontic Patients During the COVID-19 Pandemic

**DOI:** 10.3290/j.ohpd.b3957085

**Published:** 2023-03-15

**Authors:** Zhuoying Li, Ke Zhang, Yulei Huang, Manisha Pandey, Huaimin Xu, Hong Zhang

**Affiliations:** a Postgraduate Student, Department of Orthodontics, Hospital of Stomatology, Guanghua School of Stomatology, Sun Yat-sen University, Guangdong Provincial Key Laboratory of Stomatology, Guangzhou, Guangdong, People’s Republic of China. Idea, hypothesis, experimental design, performed the experiments in partial fulfillment of requirements for a degree, consulted on and performed statistical evaluation, contributed substantially to discussion, wrote and proofread the manuscript.; b Resident, Stomatological Hospital, Southern Medical University, Guangzhou, Guangdong, People’s Republic of China. Idea, hypothesis, experimental design, performed the experiments in partial fulfillment of requirements for a degree, contributed substantially to discussion, wrote and proofread the manuscript.; c Associate Professor, Department of Oral Medicine, Hospital of Stomatology, Guanghua School of Stomatology, Sun Yat-sen University, Guangdong Provincial Key Laboratory of Stomatology, Guangzhou, Guangdong, People’s Republic of China. Performed the experiments in partial fulfillment of requirements for a degree, wrote the manuscript, proofread the manuscript, consulted on and performed statistical evaluation.; d Postgraduate Student, Department of Orthodontics, Hospital of Stomatology, Guanghua School of Stomatology, Sun Yat-sen University; Guangdong Provincial Key Laboratory of Stomatology, Guangzhou, Guangdong, People’s Republic of China. Performed the experiments in partial fulfillment of requirements for a degree, consulted on and performed statistical evaluation, wrote and proofread the manuscript.; e Resident, Department of Oral and Maxillofacial Surgery, Hospital of Stomatology, Guanghua School of Stomatology, Sun Yat-sen University, Guangdong Provincial Key Laboratory of Stomatology, Guangzhou, Guangdong, People’s Republic of China. Consulted on and performed statistical evaluation, wrote and proofread the manuscript.; f Associate Professor, Department of Orthodontics, Hospital of Stomatology, Guanghua School of Stomatology, Sun Yat-sen University, Guangdong Provincial Key Laboratory of Stomatology, Guangzhou, Guangdong, People’s Republic of China. Idea, hypothesis, experimental design, contributed substantially to discussion, proofread the manuscript.

**Keywords:** COVID-19, oral health, oral hygiene, psychosocial factors, quality of life

## Abstract

**Purpose:**

To assess the levels of oral health-related quality of life (OHRQoL) in orthodontic patients both during the suspension of dental services caused by COVID-19 and after a year of dental service reinstatement, and to evaluate the associated factors for OHRQoL in those patients during the suspension period.

**Materials and Methods:**

A cross-sectional online study was conducted both during the suspension of dental service due to COVID-19 (T_1_) and after a year of dental service reinstatement (T_2_). The questionnaire – consisting of personal information, subjective complaints, OHIP-14 and oral health conditions – was completed by the participants at T_1_ and T_2_. Data were evaluated by the Χ^2^ test, the Wilcoxon rank-sum test, and multivariate logistic regression analysis.

**Results:**

324 participants were ultimately included in the study sample. The participants reported higher OHIP-14 total scores at T_1_ than T_2_ (p < 0.001). Statistically significant differences were detected in the domains psychological discomfort, psychological disability, social disability and handicap (p < 0.001). The multivariate logistic regression analysis showed that wearing fixed appliances, being over 18 years old, having delayed orthodontic treatment and poor oral hygiene habits were statistically significantly associated with higher OHIP-14 total scores at T_1_ (p < 0.05).

**Conclusion:**

The OHRQoL in orthodontic patients was negatively impacted by the suspension of dental services during COVID-19, which was reflected in all the psychosocial domains. Types of appliances, ages, delays in follow-up visits and oral hygiene habits seemed to be the factors associated with OHRQoL in orthodontic patients during the suspension.

The outbreak of coronavirus disease 19 (COVID-19), an acute respiratory infection caused by the SARS-CoV-2 virus, has alarmed people all around the world since December 2019.^4,34^ Considering the high risk of viral transmission, the Chinese government initially suspended non-urgent dental services to contain the epidemic during the early phase of COVID-19.^21^ Accordingly, routine follow-up visits and in-person treatment protocols of orthodontic patients were delayed. However, monthly orthodontic visits are particularly important for ensuring the quality of orthodontic treatments. Notably, we observed that most patients complained about their dental discomfort and delayed orthodontic treatment during the unusual large-scale suspension of dental services. Thus, the unavailability of orthodontic service and nationwide COVID-19 lockdown in the initial phase of the pandemic exacerbated negative emotions of orthodontic patients.^25,31^ Fortunately, public health services, including routine dental care, have gradually resumed with universal vaccination, since vaccines were developed to decrease of COVID-19-related hospitalisation, severity of the disease and mortality in mid-2021.^12^

Oral health-related quality of life (OHRQoL) is recognised as a multidimensional construct that corresponds to an individual’s perception of how oral health or disease impact on an individual’s daily functioning, well-being and overall quality of life.^17,20,24^ The 14-item Oral Health Impact Profile (OHIP-14), regarded as a short-form OHIP to measure OHRQoL,^10,28^ has been widely used in dental research thanks to its good psychometric properties.^1,9,32^ The most recent studies showed that chronic stress due to COVID-19 had given rise to the poorer oral health among special at-risk populations.^13^ Thus, our study aimed to assess the level of OHRQoL in orthodontic patients using OHIP-14 both during the suspension of dental services and after a year of dental service reinstatement, as well as its associated factors during the suspension.

## Materials and Methods

### Study Design

The current study was performed at the orthodontic department of the Hospital of Stomatology, Guanghua School of Stomatology, Sun Yat-sen University, PR China, from March 2020 to September 2020 and from March 2021 to September 2021. Ethical approval for this cross-sectional study was obtained from the Medical Ethics Committee of the Hospital of Stomatology, Sun Yat-sen University, PR China (approval no. KQEC-2020-23-02). Informed consent was acquired and all collected information was kept confidential.

### Study Sample

Based on the OHIP-14 total scores in our pilot trial during the suspension of services and the scores reported in a previous study prior to the COVID-19 pandemic,^33^ it was estimated that a total sample size of 315 subjects would be needed to demonstrate statistically significant differences in OHRQoL in orthodontic patients during a one-year period, with a power of 0.8 according to G-Power software (University of Düsseldorf; Düsseldorf, Germany). Considering the possible drop-out rate, 388 orthodontic patients were recruited in order to guarantee the minimum sample size required. Assessments of OHRQoL were conducted during the suspension of dental services (T_1_) and after a year of dental service reinstatement (T_2_).

Patients over the age of 14 were included if they had not returned for at least two follow-up intervals (12 weeks) at T_1_. Patients were excluded if they had non-orthodontic dental emergencies (sudden severe toothache, trauma, mouth opening restriction caused by temporomandibular disorder, shedding or breakage of dentures, loss of dental restorations, etc.) and other systemic diseases, such as pneumonia caused by the SARS-CoV-2 virus. Patients were also excluded if they had received orthodontic treatment for less than 6 months, excluding the impact of the greatest deterioration in OHRQoL in the early phase of orthodontic treatment.^16^

### Data Collection

Data were collected through an online questionnaire consisting of 4 sections. First, the characteristics of the participants were collected, i.e. gender, age, their orthodontic appliances, educational level, employment status, extraction experience (yes/no) and the time passed since the last follow-up visit. In section 2, participants were asked to provide their main subjective complaints related to orthodontic treatment. In section 3, the Chinese version of OHIP-14 was used to assess OHRQoL for satisfactory reliability and validity.^15^ The frequency of oral problems described in the 14 items was respectively determined on a five-point Likert scale (0 = never, 1 = almost never, 2 = occasionally, 3 = quite often, 4 = very often). Every two items assessed one domain of the OHRQoL. A total of seven domains were collected: functional limitation, physical pain, psychological discomfort, physical disability, psychological disability, social disability, and handicap. Higher scores represented poorer OHRQoL. Section 4 focused on oral hygiene measures and appliance-related problems.

### Statistical Analysis

SPSS software version 25.0 (IBM; Armonk, NY, USA) was used to analyse the quantitative data, setting statistical significance at p < 0.05. Descriptive data were shown as frequencies (percentages), mean ± SD, and medians (25th to 75th percentile). For the normality and homogeneity of variance of data, the Χ^2^ test and Wilcoxon rank-sum test were used to estimate the significance of differences. OHIP-14 scores were dichotomised by using median splits (25th and 75th percentiles) to assess the strength of the associations between OHRQoL and participants’ characteristics as well as oral hygiene habits at T_1_. Types of appliances, age, gender, delays in follow-up appointments, the frequency of toothbrushing and the use of dental floss or water flossers were then included as the independent factors in multivariate logistic regression analysis. The odds ratios (ORs) and 95% confidence intervals (95% CI) were calculated.

## Results

During the suspension period, 388 participants were recruited. Due to time constraints, two of them did not consent to participate in this study and another 30 participants were excluded based on the exclusion criteria. Table 1 contains an overview of the characteristics of the study sample. Of the 356 subjects who were enrolled in the study, 324 (91.0%) individuals – 72.2% adults, of which 67.6% were females – completed the OHRQoL assessments at both T_1_ and T_2_. The fixed-appliance wearers accounted for 62.0%, while clear-aligner wearers made up 38.0%. The majority of the participants (82.1%) had not attended their orthodontic visits for 3 to 5 months at T_1_.

**Table 1:** Participants’ characteristics at T_1_

Characteristics		N (%)
Age	14–18	90 (27.8)
	≥ 18	234 (72.2)
Gender	Male	105 (32.4)
	Female	219 (67.6)
Orthodontic appliance	Fixed appliance	201 (62.0)
	Clear aligner	123 (38.0)
Educational level	Junior highschool	51 (15.8)
	Senior highschool	70 (21.6)
	College	188 (58.0)
	Graduate student	15 (4.6)
Employment status	Working outside the home	71 (21.9)
	Working or studying at home	199 (61.4)
	Not working or studying yet	54 (16.7)
Extraction experience	Yes	186 (57.4)
	No	138 (42.6)
Time passed since last follow-up visit	12 to 15 weeks	126 (38.9)
	15 to 20 weeks	140 (43.2)
	Over 20 weeks	58 (17.9)

### Poorer OHRQoL in the Participants at T_1_

**Fig 1:**
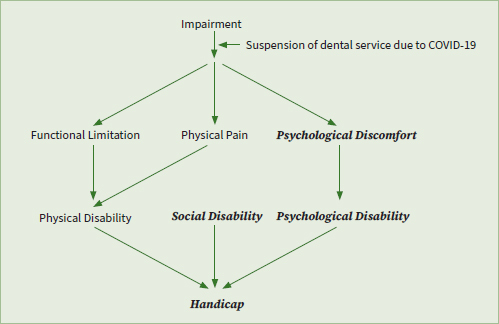
The impact of suspension of dental services due to COVID-19 on OHRQoL in orthodontic patients. Significant differences in OHRQoL in participants between T_1_ and T_2_ were found in specific domains (bold, italics).

The majority of the participants mainly complained about delayed orthodontic appointments at T_1_, while most participants had no complaints about that at T_2_ (p < 0.001, Table 2). Table 3 showed the difference in OHIP-14 scores of participants between T_1_ and T_2_. Patients reported higher OHIP-14 total scores (5.63 ± 4.50 vs 3.73 ± 1.76, p < 0.001), reflecting their worse OHRQoL at T_1_ than T_2_. Statisticaly significant differences were detected in the psychological discomfort domain (1.17± 1.48 vs 0.50 ± 0.65, p < 0.001), psychological disability domain (0.71 ± 0.91 vs 0.35 ± 0.53, p < 0.001), social disability domain (0.68 ± 0.86 vs 0.29 ± 0.50, p < 0.001) and handicap domain (0.54 ± 0.94 vs 0.29 ± 0.53, p < 0.001). Higher handicap scores at T_1_ could have developed from the aforementioned domains (Fig 1). Based on the oral health conditions listed in Table 4, participants had poorer oral hygiene habits, such as not frequently brushing teeth or using dental floss, which was more prevalent at T_1_ than T_2_ (p = 0.023, p = 0.040).

**Table 2:** Subjective complaints of participants at T_1_ and T_2_

Variables	Orthodontic patients		p-value
	T_1_ (n = 324)	T_2_ (n = 324)	
**Subjective complaints (%)**			
Delay of appointment	205 (63.3)	36 (11.1)	<0.001***
Problems with appliances	38 (11.7)	32 (9.9)	
Others	26 (8.0)	11 (3.4)	
No complaint	55 (17.0)	245 (75.6)	

*** Χ^2^ test.

**Table 3:** Mean and median OHIP-14 domain scores of participants at T_1_ and T_2_

OHIP-14 Domain	Orthodontic patients		p-value
	T_1_ (n = 324)	T_2_ (n = 324)	
OHIP-14 total score			
Mean ± SD	5.63 ± 4.50	3.73 ± 1.76	<0.001***
Median (25th, 75th)	4.50 (3.00, 7.00)	4.00 (3.00, 5.00)	
Functional limitation			
Mean ± SD	0.75 ± 1.49	0.68 ± 0.75	0.562
Median (25th, 75th)	0.00 (0.00, 1.00)	1.00 (0.00, 1.00)	
Physical pain			
Mean ± SD	0.93 ± 0.90	0.85 ± 0.81	0.235
Median (25th, 75th)	1.00 (0.00, 2.00)	1.00 (0.00, 1.00)	
Psychological discomfort			
Mean ± SD	1.17± 1.48	0.50 ± 0.65	<0.001***
Median (25th, 75th)	1.00 (0.00, 2.00)	0.00 (0.00, 1.00)	
Physical disability			
Mean ± SD	0.85 ± 1.14	0.78 ± 0.82	0.688
Median (25th, 75th)	1.00 (0.00, 1.00)	1.00 (0.00, 1.00)	
Psychological disability			
Mean ± SD	0.71 ± 0.91	0.35 ± 0.53	<0.001***
Median (25th, 75th)	0.00 (0.00, 1.00)	0.00 (0.00, 1.00)	
Social disability			
Mean ± SD	0.68 ± 0.86	0.29 ± 0.50	<0.001***
Median (25th, 75th)	1.00 (0.00, 1.00)	0.00 (0.00, 1.00)	
Handicapped			
Mean ± SD	0.54 ± 0.94	0.29 ± 0.53	<0.001***
Median (25th, 75th)	0.00 (0.00, 1.00)	0.00 (0.00, 1.00)	

*** Wilcoxon rank-sum test for paired samples.

**Table 4:** Oral health conditions of participants at T_1_ and T_2_

Variables	Orthodontic patients		p-value
	T_1_ (n = 324)	T_2_ (n = 324)	
Toothbrushing/day (%)			
≥Twice	187 (57.7)	212 (65.4)	
Occasional	112 (34.6)	101 (31.2)	
Rarely	25 (7.7)	11 (3.4)	0.023*
Use of dental floss or water flossers (%)			
Always	139 (42.9)	147 (45.4)	
Occasionally	124 (38.3)	139(42.9)	
Rarely	61 (18.8)	38 (11.7)	0.040*
Appliance-related problems (%)			
No appliance breakage	192 (59.3)	206 (63.6)	
Appliance breakage without discomfort	74 (22.8)	62 (19.1)	
Appliance breakage with slight discomfort	53 (16.4)	54 (16.7)	
Appliance breakage with obvious discomfort	5 (1.5)	2 (0.6)	0.416

* p < 0.05, Χ^2^ test.

### Associated Factors for OHRQoL at T_1_

In the multivariate logistic regression analysis, type of appliance, age, gender, delays in follow-up appointments, the frequency of toothbrushing and the use of dental floss or water flossers were included as the independent factors (Table 5). With the exception of gender, the regression analysis indicated that the factors mentioned above were statistically significantly associated with OHRQoL at T_1_. Fixed-appliance wearers showed statistically significantly poorer OHRQoL compared with clear-aligner wearers (OR: 0.577, 95% CI: 0.345–0.965, p = 0.036). Adults showed poorer OHRQoL at T_1_ (OR: 2.700, 95% CI: 1.527–4.774, p = 0.001), and more severe delays in follow-up visits were associated with statistically significantly worse OHRQoL at T_1_ (15 to 20 weeks vs 12 to 15 weeks: OR: 1.880, 95% CI: 1.100–3.216, p = 0.021; over 20 weeks vs 12–15 weeks: OR: 3.183, 95% CI: 1.574–6.436, p = 0.001). As for oral hygiene habits, those who less frequently brushed teeth or used dental floss or water flossers generally had poorer OHRQoL at T_1_ (toothbrushing, occasional vs more than twice: OR: 2.180, 95% CI: 1.291–3.680, p = 0.004; seldom vs more than twice: OR: 3.545, 95% CI: 1.345–9.342, p = 0.010; use of dental floss or water flossers, occasional vs always: OR: 1.861, 95% CI: 1.091–3.173, p = 0.023; seldom vs more than twice: OR: 2.628, 95% CI: 1.319–5.239, p = 0.006). Additionally, Table 6 showed that fixed-appliance wearers rather than clear-aligner wearers reported higher OHIP-14 scores in the physical pain, psychological discomfort, physical disability, psychological disability, social disability as well as handicap domains (p < 0.05). Furthermore, clear-aligner wearers tended to more frequently brush their teeth and use dental floss or water flossers, and reported fewer appliance-related problems, than did fixed-appliance wearers at T_1_ (p < 0.001, p = 0.031, p = 0.030).

**Table 5:** Multivariate logistic regression analysis of factors associated with OHIP-14 total scores at T_1_

Factor	B (Regression Coefficient)	OR	95% CI^a^ for OR^b^		p-value
			Lower	Upper	
Types of appliance					
Fixed appliances		1			
Clear aligners	-0.549	0.577	0.345	0.965	0.036*
Age					
14–18		1			
> 18	0.993	2.700	1.527	4.774	0.001**
Gender					
Male		1			
Female	-0.192	0.825	0.490	1.390	0.471
Time passed since last follow-up visit					
12 to 15 weeks		1			
15 to 20 weeks	0.631	1.880	1.100	3.216	0.021*
Over 20 weeks	1.158	3.183	1.574	6.436	0.001**
Toothbrushing/day (%)					
≥Twice		1			
Occasional	0.779	2.180	1.291	3.680	0.004**
Rarely	1.266	3.545	1.345	9.342	0.010*
Use of dental floss or water flossers (%)					
Always		1			
Occasional	0.621	1.861	1.091	3.173	0.023*
Rarely	0.966	2.628	1.319	5.239	0.006**

^a^CI: confidence interval; ^b^OR: odds ratio. Outcome: dichotomised OHIP-14 total scores using median split (median=5). Higher scores of OHIP-14 indicated poorer OHRQoL. *p < 0.05; ** p < 0.01.

**Table 6:** Characteristics, OHIP-14 scores and oral health conditions of participants at T_1_

Variables	Types of appliances		p-value
	Fixed appliances (n=201)	Clear aligners (n=123)	
Characteristics			
Gender (%)			
Male	66 (32.8)	39 (31.7)	
Female	135 (67.2)	84 (68.3)	0.833
Age group (%)			
14-18 years	55 (27.4)	35 (28.5)	
≥ 18 years	146 (72.6)	88 (71.5)	0.831
Educational level (%)			
Junior highschool	33 (16.4)	18 (14.6)	
Senior highschool	45 (22.4)	25 (20.3)	
College	117 (58.2)	71 (57.8)	
Graduate student	6 (3.0)	9 (7.3)	0.334
Employment status(%)			
Working outside the home	39 (19.4)	32 (26.0)	
Working or studying at home	129 (64.2)	70 (56.9)	
Not working or studying yet	33 (16.4)	21 (17.1)	0.333
Extraction experience (%)			
Yes	120 (59.7)	66 (53.7)	
No	81 (40.3)	57 (46.3)	0.286
Time passed since last follow-up visit (%)			
12 to 15 weeks	70 (34.8)	56 (45.5)	
15 to 20 weeks	89 (44.3)	51 (41.5)	
Over 20 weeks	42 (20.9)	16 (13.0)	0.080
OHIP-14 scores			
OHIP-14 total score			
Mean ± SD	6.63 ± 4.80	3.98 ± 3.41	
Median (25th, 75th)	6.00 (4.00, 8.00)	3.00 (1.00, 5.00)	<0.001***
Functional limitation			
Mean ± SD	0.84 ± 1.52	0.61 ± 1.45	
Median (25th, 75th)	0.00 (0.00, 1.00)	0.00 (0.00, 1.00)	0.070
Physical pain			
Mean ± SD	1.0 ± 0.83	0.80 ± 0.99	
Median (25th, 75th)	1.00 (0.00, 1.00)	0.00 (0.00, 2.00)	0.001**
Psychological discomfort			
Mean ± SD	1.36 ± 1.50	0.86 ± 1.40	
Median (25th, 75th)	1.00 (0.00, 2.00)	0.00 (0.00, 2.00)	<0.001***
Physical disability			
Mean ± SD	1.10 ± 1.30	0.45 ± 0.63	
Median (25th, 75th)	1.00 (0.00, 2.00)	0.00 (0.00, 1.00)	<0.001***
Psychological disability			
Mean ± SD	0.81 ± 1.00	0.54 ± 0.70	
Median (25th, 75th)	1.00 (0.00, 1.00)	0.00 (0.00, 1.00)	0.048*
Social disability			
Mean ± SD	0.85 ± 0.95	0.39 ± 0.60	
Median (25th, 75th)	1.00 (0.00, 1.00)	0.00 (0.00, 1.00)	<0.001***
Handicap			
Mean ± SD	0.67 ± 1.10	0.33 ± 0.54	
Median (25th, 75th)	0.00 (0.00, 1.00)	0.00 (0.00, 1.00)	0.022*
Oral health conditions			
Toothbrushing/day (%)			
≥Twice	109 (54.2)	78 (63.4)	
Occasionally	84 (41.8)	28 (22.8)	
Rarely	8 (4.0)	17 (13.8)	<0.001***
Use of dental floss or water flossers (%)			
Always	82 (40.8)	57 (46.3)	
Occasionally	71 (35.3)	51 (41.5)	
Rarely	48 (23.9)	15 (12.2)	0.031*
Appliance-related problems (%)			
No appliance breakage	109 (54.2)	83 (67.5)	
Appliance breakage without discomfort	50 (24.9)	24 (19.5)	
Appliance breakage with slight discomfort	37 (18.4)	16 (13.0)	
Appliance breakage with obvious discomfort	5 (2.5)	0 (0.0)	0.030*

* p < 0.05; ** p < 0.01; *** p < 0.001. The p-values for the characteristics and oral health conditions were determined using the Χ^2^ test. The p-values for the OHIP-14 scores were determined using the Wilcoxon rank-sum test for two independent samples.

## Discussion

This cross-sectional study was conducted to assess the impact of the large-scale suspension of dental services on OHRQoL in orthodontic patients during the COVID-19 pandemic. In this study, the overall and domain-specific OHIP-14 scores of participants were considerably higher at T_1_ than T_2_, indicating a disruptive impact of suspension on OHRQoL in orthodontic patients during COVID-19. In particular, adult fixed-appliance wearers, who experienced delayed orthodontic treatment and had worse oral hygiene habits, were more negatively impacted.

OHRQoL, a multidimensional concept, assesses physical, psychological, and social aspects.^8^ In our study, the OHRQoL in participants was worse at T_1_ than T_2_ over all the psychological and social domains (Table 3). Notably, the OHRQoL in participants was poorer even in the handicap domain at T_1_, which was rarely reported in previous studies. David Locker, who first proposed the framework measuring oral health, regarded handicap as the outcome that had the most disruptive impact on people’s lives in OHIP-14.^17,18^ We speculated that the COVID-19 pandemic with the severly altered social environment it caused, might serve as the source of anxiety to exacerbate the discomfort of orthodontic patients at T_1_ (Fig 1). Similarly, a recent study has shown that the specific COVID-19 prevention measures represented a significant threat to the quality of life in cancer patients.^7^ Otherwise, consistent with the study by Guo et al^11^ on orthodontic patients during COVID-19, our results showed that delayed appointments were the most common concern of orthodontic patients during the suspension caused by COVID-19 (Table 2). Moreover, our data showed a gradual decrease in OHRQoL with the extension of the delay in follow-up visits (Table 5). We suggest that increasing anxiety over delayed orthodontic appointments could have given rise to the negative psychosocial impact on the OHRQoL in orthodontic patients during COVID-19. As a whole, our data revealed that the participants’ daily life was negatively impacted by the suspension of dental services during COVID-19, especially in terms of the psychosocial domain.

Interestingly, most studies have suggested that routine orthodontic appointments helped motivate patients to develop better oral hygiene habits, which were associated with better OHRQoL.^6,19^ In this study, participants had worse oral conditions (Table 4) compared with that reported in the previous study.^3^ We believe that it could be attributed to the lack of routine oral care provided by dentists during COVID-19. As a result, the poor oral conditions led to patients’ worse OHRQoL in the physical domains during COVID-19 compared to the pre-pandemic situation.^2^ Thus, preventive oral hygiene measures could improve their OHRQoL and, according to some authors, even play a role in preventing COVID-19 infections and severe complications.^29^

In this study, the participants wore orthodontic appliances including fixed appliances and clear aligners, since they were all undergoing comprehensive orthodontic treatment. OHRQoL in fixed-appliance wearers was poorer than that of clear-aligner wearers at T_1_ (Table 5; Table 6), in agreement with previous studies on the difference of OHRQoL between them.^22,30^ Cotrin et al^5^ reported that the breakage of fixed appliances was quite common during the pandemic. Similarly, our results indicated that fixed-appliance wearers had more severe appliance-related problems, giving rise to their poorer OHRQoL (Table 6). Otherwise, fixed-appliance wearers generally had worse oral hygiene practices at T_1_ (Table 6), which could account for the difference of OHRQoL between the two groups.

In addition to fixed-appliance wearers, the participants over 18 years old had poorer OHRQoL, as shown in the multivariate logistic regression analysis (Table 5). A cross-sectional study indicated that adult orthodontic patients experienced worse OHRQoL than teenage patients, particularly in the psychological domains.^23^ Adults were reported to have stronger aesthetic needs but poorer adaptability to the orthodontic appliances, which may aggravate their anxiety level.^26^ Recent studies on COVID-19 have found that adults tended to experience more stress, anxiety and depression, whereas there was a decrease in the perception of oral health problems among adolescents.^14,27^ Hence, adult patients might require additional attention during public health emergencies.

Some limitations of the present study should be considered. Patients under the age of 14 were excluded due to the applicability of OHIP-14 scale, which might limit the usability of the findings, as the sample is not actually representative of the general public. In addition, our study was limited to analysis of a study population in one region, which may vary due to different social factors, such as policies for epidemic prevention, races, cultures, etc. The OHRQoL in orthodontic patients of different sociocultural backgrounds could be evaluated in future studies.

## Conclusions

This study highlights the negative impact of the suspension of dental services during COVID-19 on the OHRQoL in orthodontic patients in all the psychosocial domains. Type of appliance, age, delays in follow-up visits and oral hygiene habits could be the associated factors for OHRQoL in orthodontic patients during the suspension of dental services. It is helpful for dentists to provide considerate and personalised service to patients from a psychosocial perspective, also and especially during public health emergencies.
